# Layers, broiler chickens and their F1 cross develop distinctly different caecal microbial communities when hatched and reared together

**DOI:** 10.1111/jam.15558

**Published:** 2022-04-18

**Authors:** Nicky‐Lee Willson, Robert J. Hughes, Philip I. Hynd, Rebecca E. A. Forder

**Affiliations:** ^1^ School of Animal and Veterinary Sciences The University of Adelaide Roseworthy South Australia Australia; ^2^ Formerly with the South Australian Research and Development Institute (SARDI) Pig and Poultry Production Institute (PPPI) Roseworthy South Australia Australia

**Keywords:** broiler, layer, caecal microbiota, F1 cross, chicken

## Abstract

**Aim:**

To compare the caecal microbiota of layer, broiler, and intermediate F1 layer × broiler cross birds with the hypothesis that significant differences in caecal microbial composition would persist between the three groups when host and environmental interactions were minimized.

**Methods and Results:**

Caecal contents were characterized using 16S rRNA for males of broiler (*n* = 12), layer (*n* = 12) and F1 layer × broiler cross (*n* = 9) birds that were hatched and reared under the same conditions. The microbial community structure differed significantly between the three groups of birds at phylum, genus and OTU levels, with clear separation of the groups observed. *Firmicutes* was the phylum most represented across samples; however, the high abundance of *Proteobacteria* in the layer birds at d28 post‐hatch was unexpected, and driven by a higher abundance of *E. coli*.

**Conclusions:**

The microbiota phylotype between broilers, layers and their F1 cross significantly differed in community structure, diversity and relative abundance in the absence of environmental confounding, which is generally difficult to avoid in microbial studies.

**Significance and Impact of Study:**

The results provide a unique comparison and evidence that there is a strong genetic component driving microbial composition within poultry strains, despite the embryonic development occurring *in ovo*.

## INTRODUCTION

Globally, there are now hundreds of distinct breeds of domestic chickens (*Gallus gallus domesticus*), which have been adapted (via genetic selection) into major food sources specialized for egg production (layers) or rapid growth (broilers) (Qanbari et al., 2019). From the middle of the twentieth century, divergent selection for production traits has included high growth, carcass yield and increased feed efficiency for broilers and increased egg production and feed efficiency for layers (Druyan, 2010). The resultant gains are enormous for each industry. Bodyweight increases in excess of 400%, and a reduction of 50% in feed conversion ratio has been achieved in the chicken meat industry (Zuidhof et al., 2014). In the layer industry, most modern commercial layers will now produce in excess of 300 eggs per annum, while their ancestor the Red Jungle Fowl generally produces a single clutch of 5–9 eggs per year (Qanbari et al., 2019). There are many factors influencing the performance of both broiler and layer birds, with the intestinal microbiota regarded as highly influential (Qi et al., 2019; Stanley et al., 2014).

Direct comparisons of the microbiota between both layers and broilers raised under similar environmental conditions are sparse. Roman Laying Hens and Arbor Acres broiler chickens were compared at 120 days of age with both breeds hatched, raised and fed identically (Qi et al., 2019). The authors reported distinct microbial communities with greater bacterial diversity in the layer hens. Qi et al. (2019) investigated the metagenomes of two samples and found an increase in abundance of functions related to carbohydrate metabolism, lipid metabolism, amino acid metabolism, as well as glycan biosynthesis and metabolism in the layer hens compared to broilers, indicating the influence of microbial metabolites on the performance of each breed is likely significant. Another study by Schokker et al. (2015) examined the composition of the jejunal microbiota between two genetically divergent broiler lines (hatched and reared together) that are known to differ in immunological infection response, and found similar diversity, but differing composition. Transcriptomic analysis of the jejunum in these birds showed greater differences in cell cycle regulation and apoptosis clusters, than changes in immunity. That result however may differ in a more immunologically active region of the gut such as the distal ileum (including caecal tonsils) and gut associated lymphoid tissue, but the differing microbial composition and concurrent differences in cell cycle regulation are of interest.

Willson et al. (2017, 2018) utilized broiler and layer strains of poultry to investigate biological factors contributing to growth variation in poultry. RNA sequencing of broilers, layers and progeny from a F1 layer × broiler cross‐revealed highly divergent hepatic transcriptomes, particularly between the layer and broiler birds (Willson et al., 2018). Differentially expressed genes, particularly those related to cell cycle regulation and insulin signalling, were enriched to the FoxO signalling pathway. Significant gene ontology terms included ‘positive regulation of glucose import’ and ‘cellular response to oxidative stress’, which are also consistent with FoxOs regulation of glucose metabolism. Previous investigation of the three groups of birds also demonstrated differential carcass fatty acid composition, and an overall increase in fatty acid metabolism in the broilers, despite all birds being raised in the same environment and fed identical diets (Willson et al., 2017).

Nutrition as a factor in shaping the host′s intestinal microbiota, as well as functional modulation of host genes associated with nutrient‐signalling pathways, including: insulin/insulin‐like growth factor 1 signalling, and the FoxO signalling pathways, are well established (Kim & Jazwinksi, 2018). Microbial production of short‐chain fatty acids is also known to affect host metabolism, including lipid and glucose metabolism (Besten et al., 2013), alterations to all of which we have previously observed in the broiler, layer and F1 layer × broiler birds (Willson et al., 2018). Given our three growth phenotypes were all fed the same diet, we concluded that the differential expression of hepatic genes associated with key nutrient pathways that we have previously discovered, may be driven by microbial differences between the strains.

Comparisons of microbiota between studies are hindered by a multitude of both host and environmental factors including study location, age, gut region, sex, diet, housing, hygiene, medications, temperature, litter, breed and maternal factors (Kers et al., 2018). The current study was designed to investigate the caecal microbial populations in broiler, layer and an F1 layer × broiler cross produced from the same parent stock that were hatched and reared together to minimize the effects of host and environmental factors.

## MATERIALS AND METHODS

### Birds and management

The Animal Ethics Committee of The University of Adelaide (approval #S‐2015‐171) and PIRSA (approval #24/15) approved all procedures involving the use of animals.

The samples used in the current study were collected from an animal trial previously conducted and described by Willson et al. (2017). In brief, 150 newly hatched male chicks were obtained from the HiChick Breeding Company Pty. Ltd.. Birds were of three strains: Broiler (Broiler Breeder Line; *n* = 50), Layer (Isa Brown; *n* = 50) and an F1 cross (*n* = 50), produced using Isa Brown roosters and Broiler Line breeder hens. Chicks were separated by strain and reared in a six unit‐rearing pen (25 birds per pen/*n* = 2 pens per strain), in a temperature‐controlled facility at the SARDI PPPI Poultry Research Unit, Roseworthy Campus, The University of Adelaide. The facility temperature was maintained at 33°C for the first 3 days, then gradually reduced to 21°C by d28. All birds were fed a commercial broiler starter diet (no added antimicrobials or coccidiostats), and had unrestricted access to water provided via nipple drinker lines. Weekly bodyweights and feed intake were recorded to monitor bird performance and have been previously presented by Willson et al. (2018). At d28 post‐hatch birds were euthanized by cervical dislocation. Caecal contents were collected and stored at −20°C prior to DNA extraction. Utensils were cleaned with ethanol between samples to avoid inter‐sample contamination.

### DNA extraction, amplification and sequencing

Total DNA was extracted from ~200 mg of caecal samples collected from broiler (*n* = 12), layer (*n* = 12), and F1 layer × broiler cross (*n* = 9) birds, using a QiAamp Fast DNA mini stool kit (Qiagen). The V3‐V4 region of the 16S rRNA was amplified and sequenced using forward primer 5′CCTAYGGGRBGCASCAG3′ and reverse primer 5′GGACTACNNGGGTATCTAAT3′, incorporating barcode sequences and capture sequences for MiSeq sequencing (Illumina MiSeq; 2 × 300 bp). Sequencing was performed by the Australian Genomes Research Facility (AGRF).

Forward and reverse reads were assembled using PEAR, version 0.9.5 (Zhang et al., 2014). Sequence data were analysed in QIIME version 1.8.4 (Caporaso et al., 2010) using default parameters, USEARCH version 8.0.1623 and UPARSE (Edgar, 2010). All 33 samples were successfully sequenced giving 1,789,839 raw sequences with 1,492,795 remaining following quality control. Average sequences obtained for each strain were broiler 42,734.27 ± 3342.12, cross 45,446.00 ± 5706.41 and layer 47,354.83 ± 2856.00. Sequences were quality filtered and full‐length duplicate sequences removed and sorted by abundance. Singletons or unique reads in the data set were discarded. Sequences were clustered and chimeric sequences were filtered using the ‘rdp_gold’ database as a reference. The number of reads in each OTU was obtained by mapping reads back to OTUs with a minimum identity of 97%. Taxonomy was assigned using QIIME version 1.8.4 defaults and the GreenGenes database (DeSantis et al., 2006).

### Statistical analysis

Statistical analysis and visualization of 16S rRNA sequence data were performed in Calypso (Zakrzewski et al., 2017) using square root transformed data combined with total sum scaling (TSS) method (Hellinger Transformation) (Legendre & Gallagher, 2001). Beta diversity statistics were calculated using Adonis (Bray‐Curtis distance), alpha diversity metrics were calculated using Shannon Index, richness and evenness, and the linear discriminant analysis (LDA) effect size method (LEfSe) was used to identify significant taxa differentially associated with each strain. Inter‐ and intra‐sample variation comparisons were conducted by analysis of similarities (ANOSIM) and ordination analysis was conducted using redundancy analysis (RDA). Calypso (Zakrzewski et al., 2017) scripts use standard parametric and non‐parametric tests to compare the abundance of taxa using tests specifically developed for counts data (DEseq2, ANCOM and ALDEx2). The univariate feature was used to compare taxonomic abundance between broiler, layer and cross samples (FDR <0.05) with a Tukey′s post hoc. Correlations between bodyweight data and microbiota were tested by Pearson′s correlations.

## RESULTS

### Community structure

**FIGURE 1:**
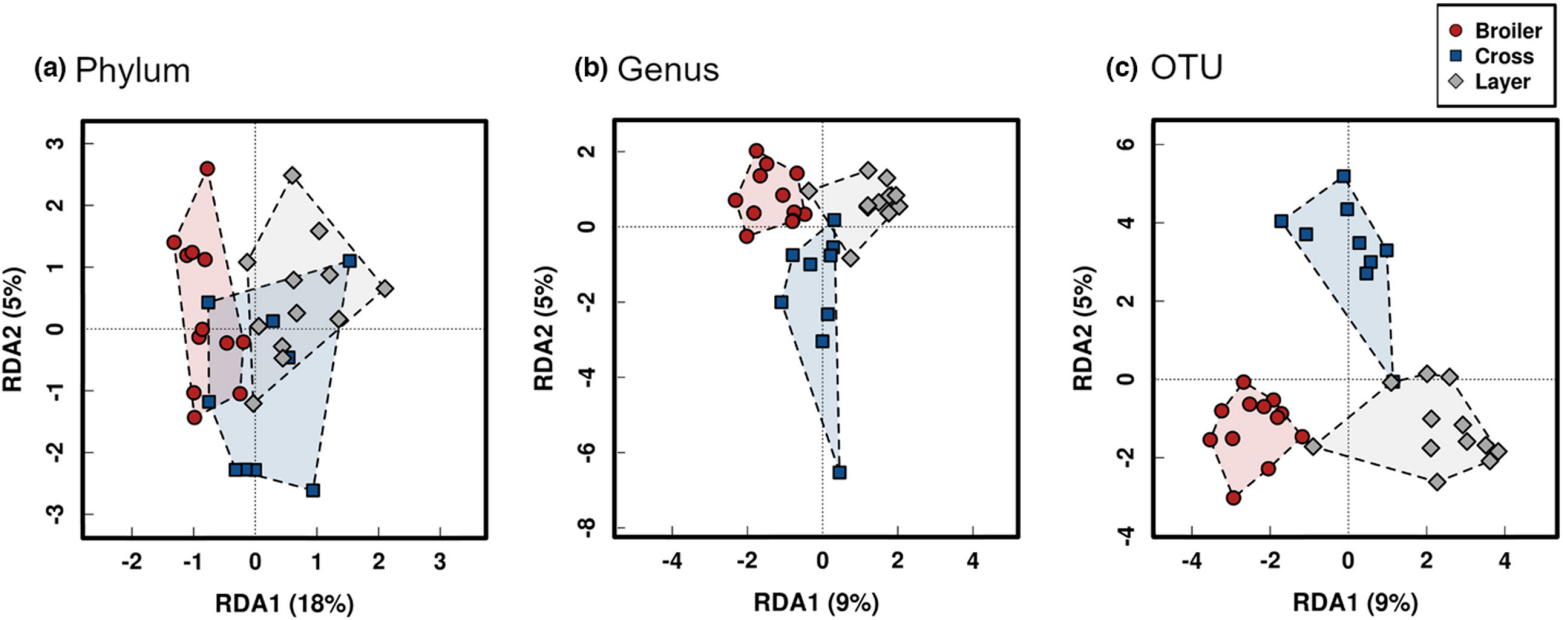
Redundancy analysis (RDA) plot demonstrating microbial community separation between broiler (red), cross (blue) and layer (grey) caecal samples at the level of (a) phylum (b) genus and (c) OTU

The community structure was analysed by Adonis, using Bray–Curtis distance. Comparisons between broiler, cross and layer birds revealed significant differences in caecal community structure between the three groups at the levels of phylum (*p* = 6.66e‐04), genus (*p* = 3.33e‐04) and OTU (*p* = 3.33e‐04). Comparisons remained significant at each described level between broiler vs. layer, broiler vs. cross and layer vs. cross, with the exception of the latter in which differences in community structure were not detected between layer and cross birds at the level of phylum (*p* = 0.206). The community separation between the three strains is further evidenced by the redundancy analysis (RDA) plot at the levels of phylum (*p* = 0.001; Figure 1a), genus (*p* = 0.001; Figure 1b) and OTU (*p* = 0.001; Figure 1c). In conjunction with the Adonis results, the RDA was not significant between layer vs. cross birds at the level of phylum (*p* = 0.058), but remained significant for all other paired comparisons at phylum, genus and OTU levels (data not shown). Analysis of similarity (ANOSIM) using Bray–Curtis distance was used to assess the caecal sample‐to‐sample dissimilarity between broiler, cross and layer birds. The dissimilarity matrix was significant at the OTU level between broiler vs. layer (*R* = 0.596; *p* = 0.001), broiler vs. cross (*R* = 0.159; *p* = 0.028) and layer vs. cross (*R* = 0.284; *p* = 0.003).

### Diversity analyses

**FIGURE 2:**
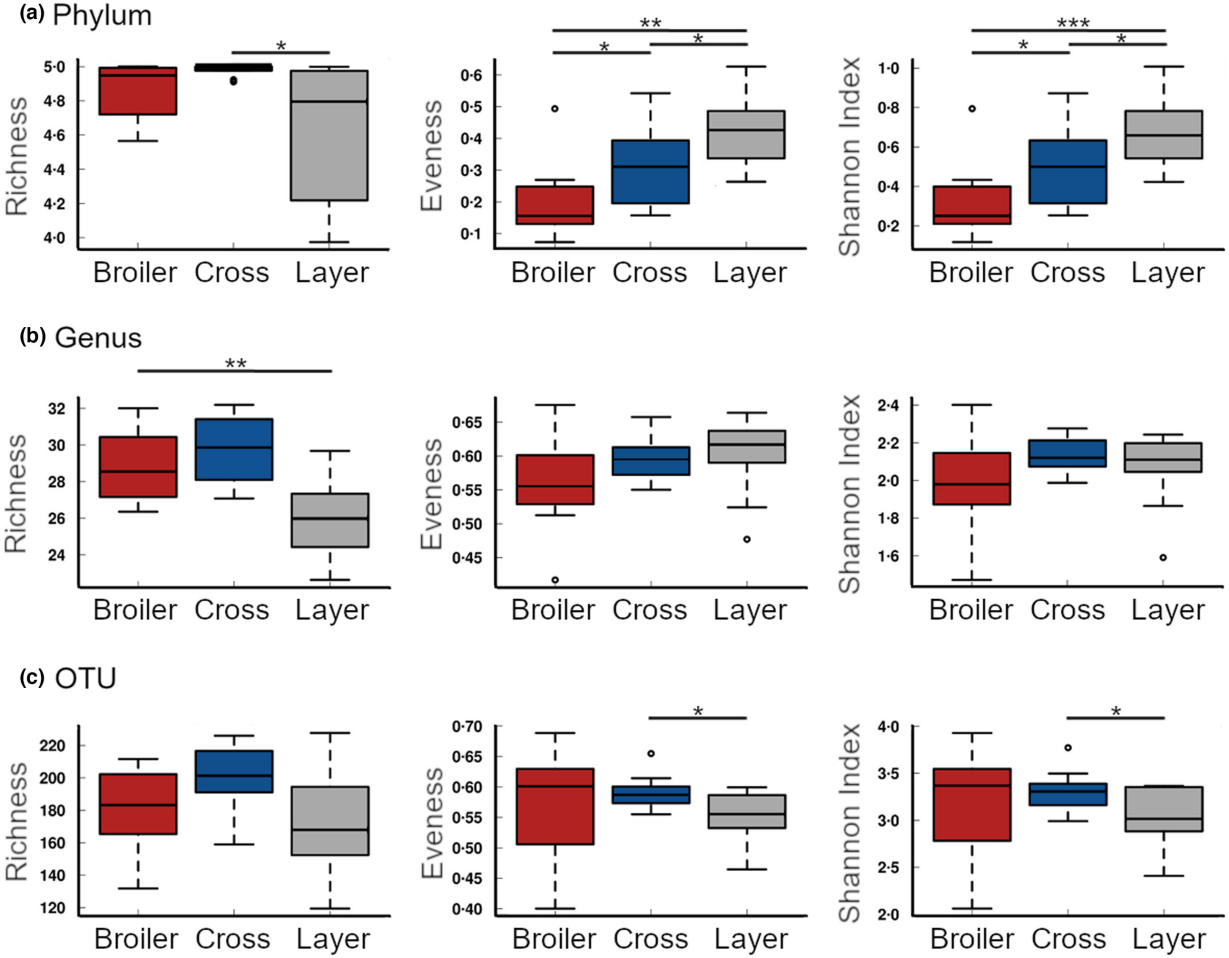
Alpha diversity statistics (richness, evenness and Shannon index) for broiler, layer and cross caecal samples compared at the taxonomic levels of (a) phylum (b) genus and (c) OTU. *p < 0.05, **p < 0.01, *** *p* < 0.001

At the level of phylum, significant differences were detected between broiler, layer and cross samples for evenness (*p* = 1.5e‐04), and Shannon Index (*p* = 2.1e‐04; Figure 2a). Richness was significantly different however only between cross and layer birds (*p* = 0.012). At the level genus, richness was highest in the cross samples and significantly different between broilers and layers (*p* = 0.003), with no differences detected for evenness or Shannon Index between the three strains (Figure 2b). Layer caecal samples had the lowest richness values for all three taxonomic levels, and the highest evenness with the exception of OTU. Cross and layer samples significantly differed at OTU level for both evenness (*p* = 0.03) and Shannon Index (*p* = 0.045; Figure 2c).

### Abundance

**FIGURE 3:**
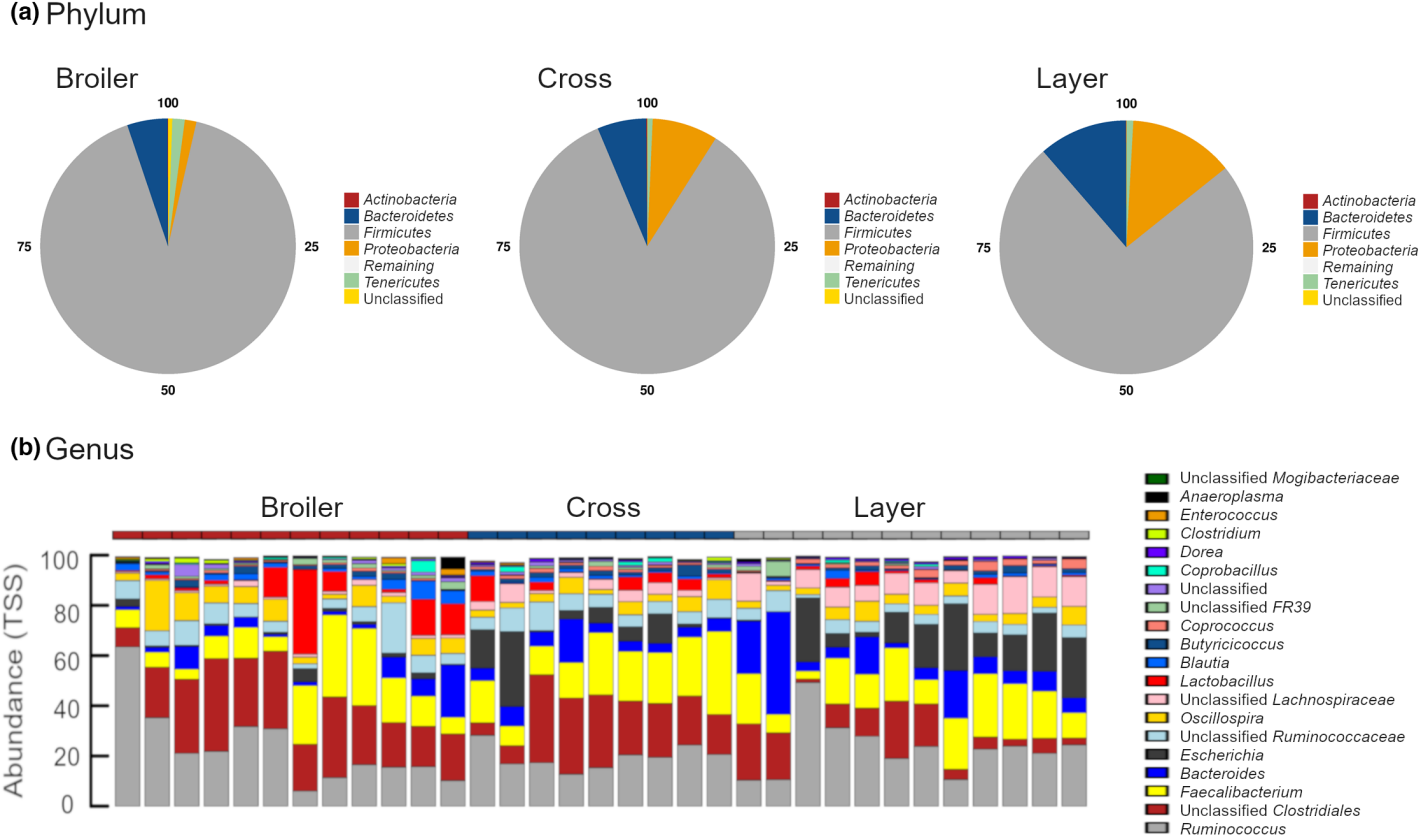
Relative abundance (%) of taxa in caecal samples collected from broiler, cross and layer birds for the taxonomic levels of (a) phylum and (b) genus

*Firmicutes* were the dominant phylum present in all samples (Figure 3a), most abundant in broilers (91.25%) and lowest in layers (74.37%; *p* = 0.001). The second most abundant phyla in both layer (13.39%) and F1 cross birds (8.31%) samples were *Proteobacteria*, while broilers had the lowest levels at 1.47% (broiler vs. layer, *p* = 0.002). *Bacteroidetes* was the second most abundant phylum in broiler samples at 5.11%. F1 cross birds (0.11%) had significantly higher levels of *Actinobacteria* compared with broilers (0.05%; *p* = 0.009) and layers (0.06%; *p* = 0.035). Low levels of unclassified taxa were detected for broiler (0.54%), layer (0.03%) and cross (0.04%) samples.

*Ruminococcus* was the most abundant genus, which on average accounted for 21.94% of total relative abundance across samples, followed by Unclassified *Clostridiales* (17.98%), *Faecalibacterium* (16.31%), *Escherichia* (7.71%), *Bacteroides* (7.54%), Unclassified *Ruminococcaceae* (5.97%) *Oscillospira* (5.25%), Unclassified *Lachnospiraceae* (4.51%), *Lactobacillus* (3.95%) and *Blautia* (1.5%; Figure 3b). There were eight taxa that differed in relative abundance with an FDR <0.05, Table 1, most significant of which was Unclassified *Lachnospiraceae*. Four of the eight differentially abundant taxa were significant between broiler and layer birds only, including *Coprococcus, Escherichia, Enterococcus,* and *Clostridium,* with intermediate non‐significant abundance of these taxa in the cross caecal samples.

**TABLE 1:** Eight differentially expressed taxa at the level genus between broiler, F1 cross and layer birds. Values are mean relative abundance percent (± SEM)

Genus	Broiler	Cross	Layer	*p*‐value	FDR (<0.05)
Unclassified *Lachnospiraceae*	1.22 ± 0.18 ^a^	3.38 ± 0.69 ^b^	8.91 ± 1.05 ^c^	<0.001	< 0.001
*Coprococcus*	0.64 ± 0.08 ^a^	1.12 ± 0.12 ^ab^	1.94 ± 0.33 ^b^	<0.001	0.013
*Staphylococcus*	0.04 ± 0.01^a^	0.46 ± 0.18 ^b^	0.05 ± 0.01^a^	0.001	0.021
Unclassified *Clostridiales*	23.21 ± 2.40^a^	20.51 ± 3.38 ^a^	10.23 ± 2.31^b^	0.002	0.026
*Escherichia*	1.47 ± 0.43^a^	8.30 ± 3.19 ^ab^	13.38 ± 2.90 ^b^	0.003	0.027
*Enterococcus*	0.63 ± 0.24^a^	0.20 ± 0.08 ^ab^	0.03 ± 0.01 ^b^	0.003	0.027
*Clostridium*	0.65 ± 0.15^a^	0.37 ± 0.13 ^ab^	0.15 ± 0.04 ^b^	0.004	0.027
*Candidatus_Arthromitus*	0.04 ± 0.01^a^	0.00 ± 0.00 ^b^	0.01 ± 0.01 ^b^	0.007	0.048

^a,b,c^Differing superscripts denotes significance at *p* < 0.05 within row.

There were 62 OTUs significantly different in abundance with an FDR <0.05. MegaBlast was used to assess the OTUs as a reference for supplementary annotation and yielded limited results on the top hits. Of these, significant overlap in results was detected including 28 OTUs identified as ‘uncultured bacterium’ ranging from 91.83% to 100% sequence identity, 10 OTUs identified as uncultured *Ruminococcaceae* bacterium ranging from 96.31–100% sequence identity and seven OTUs identified as uncultured *Lachnospiraceae* bacterium ranging from 98.51% to 100% sequence identity. There were five OTUs identified at 100% sequence identity across the amplified region to specific strains within the current study, Table 2, of which the most abundant was highly similar to *Escherichia Coli* in layer caecal samples.

**TABLE 2:** MegaBlast results for five differentially expressed OTUs with 100% sequence identity species match across the amplified region between broiler, cross and layer birds at an FDR <0.05. Values are mean relative abundance percent (± SEM)

OTU ID	MegaBlast ID	Broiler	Cross	Layer	*p*‐ value	FDR (<0.05)
OTU_28	*Enterococcus faecium* strain OV3‐6	0.63 ± 0.24^a^	0.2 ± 0.08^ab^	0.03 ± 0.01^b^	0.003	0.019
OTU_3	*Escherichia coli* strain 1500	1.47 ± 0.43^a^	8.30 ± 3.19 ^ab^	13.38 ± 2.90 ^b^	0.003	0.017
OTU_249	*Lachnospiraceae* bacterium KGMB03038	0.01 ± 0.00^a^	0.03 ± 0.01^a^	0.07 ± 0.01^b^	<0.001	<0.001
OTU_233	*Mordavella massiliensis*	0.04 ± 0.01^a^	0.14 ± 0.03^a^	0.30 ± 0.05^b^	<0.001	<0.001
OTU_63	*Staphylococcus saprophyticus*	0.04 ± 0.01^a^	0.43 ± 0.17^b^	0.05 ± 0.01^a^	0.001	0.008

^a,b^Differing superscripts denoted significance at *p* < 0.05 within row.

**FIGURE 4:**
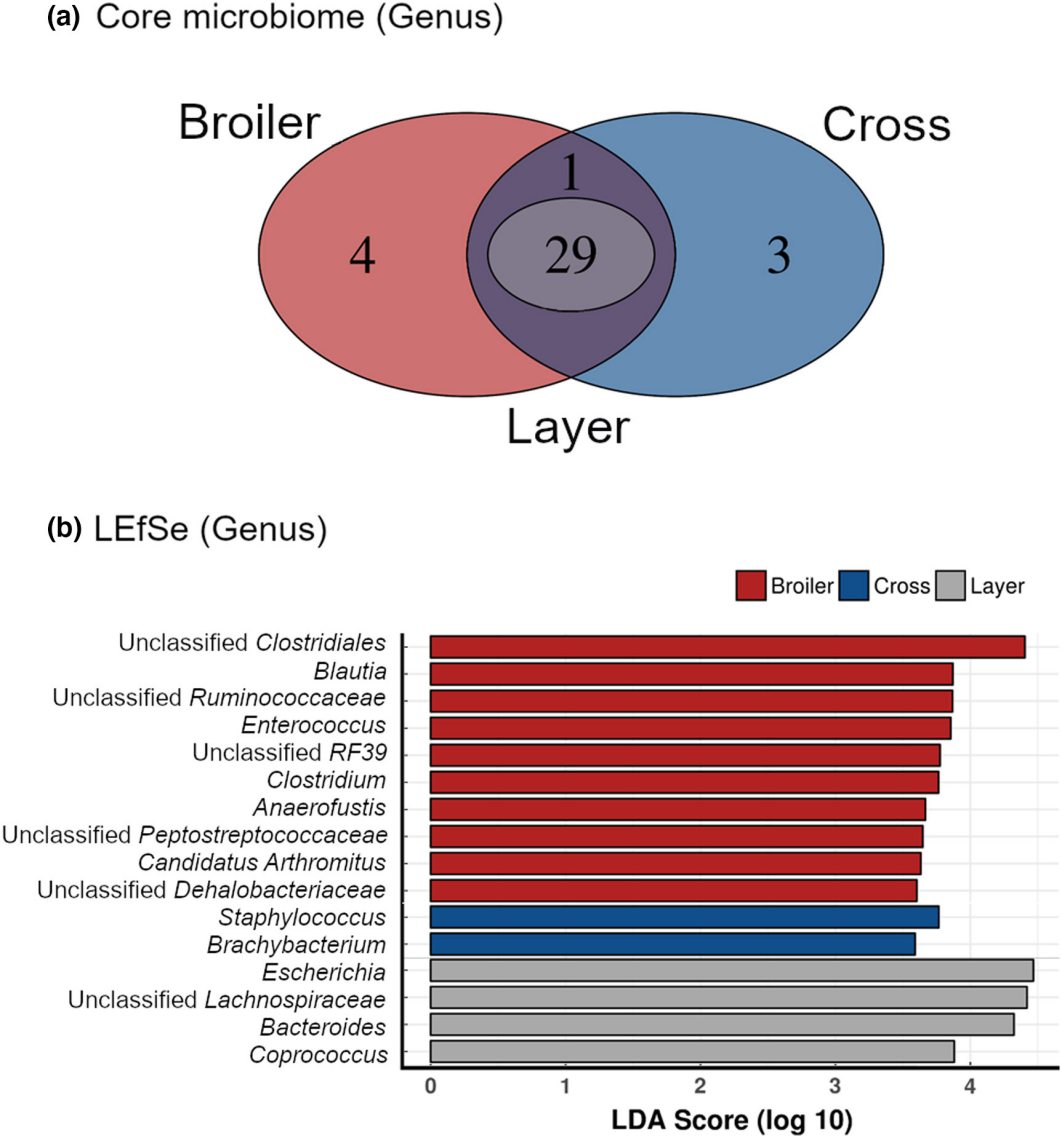
(a) Venn diagram illustrating unique and shared taxa (genus) between broiler (red), cross (blue) and layer (purple) caecal samples, and (b) differentially abundant taxa (genus) identified by linear discriminant analysis effect size (LEfSe) enriched for broiler (red), cross (blue) and layer (grey) samples

The common microbiota was assessed at the level of genus. This revealed 29 taxa in common between the three groups, four unique to broilers, three unique to the F1 cross birds and one unique to samples of layer origin (Figure 4a). Taxa that were significantly enriched to each strain were investigated using linear discriminant analysis (LDA) effect size method (LEfSe). There were 10 different genera significantly enriched to broilers with LDA scores ranging between 3.60 and 4.41 with unclassified *Clostridiales* being the highest. Two genera were enriched in cross samples, *Staphylococcus* (LDA score 3.77) and *Brachybacterium* (LDA score 3.59). There were four genera significantly enriched in the layer samples with *Escherichia* being the most significant with an LDA score of 4.47.

### Bodyweight and microbiota correlations within strain

**FIGURE 5:**
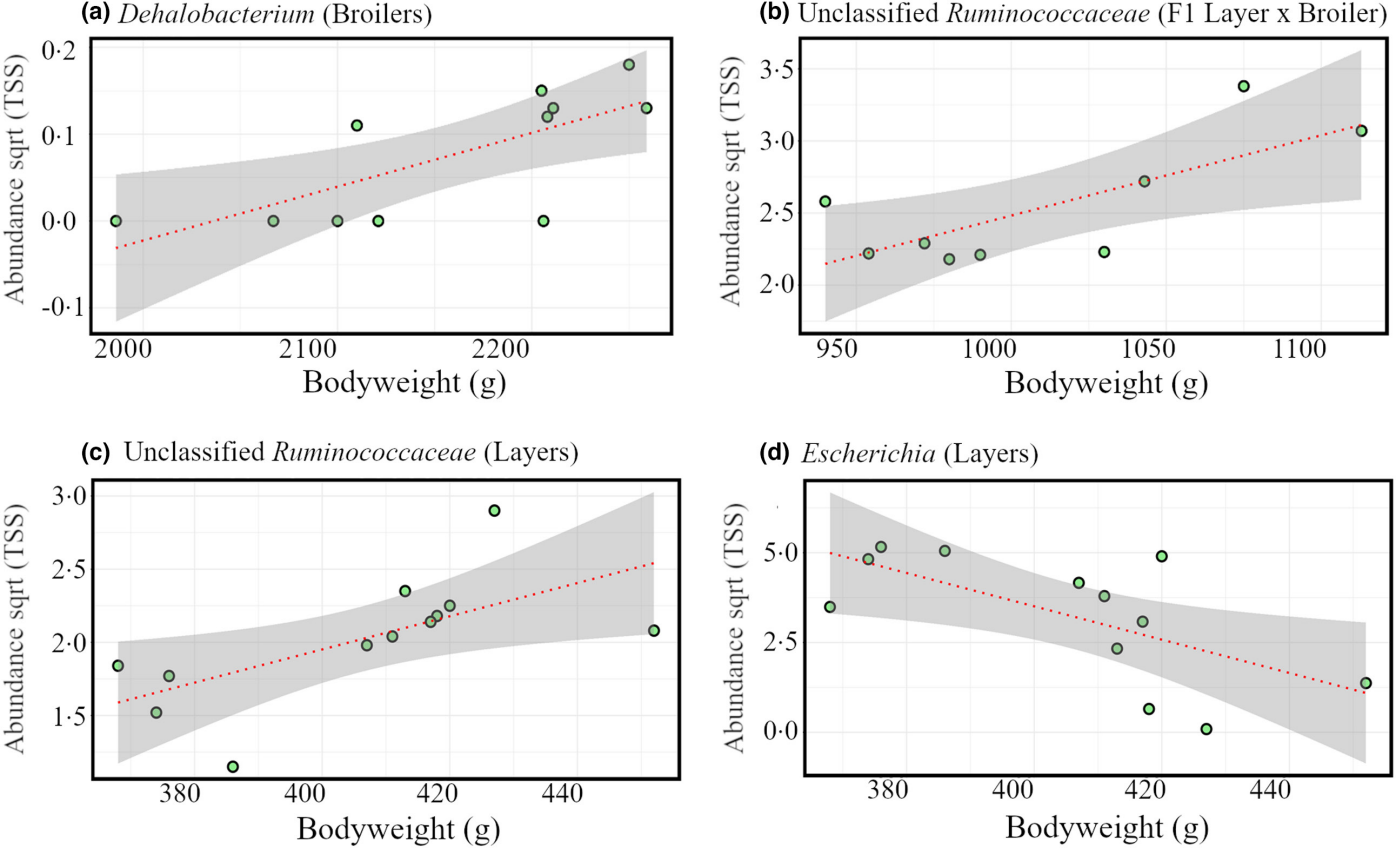
Pearson′s correlations between bodyweight (g) and genus. Correlations were conducted for each group, with significant (*p* < 0.05) correlations presented for (a) broilers, (b) F1 layer × broiler cross, and (c, d) layer birds

Pearson′s correlations were tested within each group to determine relationships with bodyweight at the level genus. *Dehalobacterium* in the broiler caecal samples was positively correlated (*R* = 0.72; *p* = 0.012) with bodyweight, Figure 5a. Unclassified *Ruminococcaceae* were positively correlated with bodyweight in both F1 Layer × Broiler cross caecal samples (*R* = 0.74; *p* = 0.021, Figure 5b) and layer caecal samples (*R* = 0.65; *p* = 0.022, Figure 5c). *Escherichia* were negatively correlated with bodyweight in layers (*R* = −0.66; *p* = 0.021, Figure 5d).

## DISCUSSION

For the past 10 years, the advancement and understanding of the composition of poultry intestinal microbiota have been significant. There remains however a high degree of variation in the overall structure of microbiota observed between different studies, which was highlighted by Stanley et al. (2013), and remains a current comparative complication. Comparisons of microbiota between studies are hindered by a multitude of both host and environmental factors including: study location, age, gut region, sex, diet, housing, hygiene, medications, temperature, litter, breed and maternal influence (Kers et al., 2018). The current study design accounts for these factors with the exception of maternal influence, which we discuss further. It was hypothesized that significant differences in caecal microbial composition would remain between the three strains after the minimisation of host (by using genetically related F1 intermediates) and environmental confounding.

Culture independent methods (such as sequencing), to explore the intestinal microbiota, are dispelling the notion that the embryonic intestinal tract is sterile and colonization only occurs post‐hatch. This indicates that the hen may influence the development of progeny microbiota despite the embryo developing *in ovo* and the absence of placental‐like effects as seen in mammals. Evidence to support maternal effects in chickens includes the transfer of maternal antibodies to chicks (Hamal et al., 2006), and increasing evidence to suggest transgenerational effects on the growth and performance of chicks in response to the hen′s physiological status. For example, broiler breeder hens are reared under a commercial management practice of feed restriction in order to prevent excess fat deposition and fertility loss due to their genetic potential for rapid growth. This practice however is associated with chronic stress, which has been shown to cause sex‐dependent alterations in progeny for growth rate, heterophil:lymphocyte ratio and response to LPS challenge (Hynd et al., 2016). Ho et al. (2011) showed that for the traits they measured, the yolk source as opposed to embryo genotype was more influential in influencing embryonic development, elevating the importance of the maternal environment in poultry.

Deeming (2005) found microbiota in the yolk of multiple healthy avian species, while Pedrosa (2009) used molecular and microscopic techniques and found microbial populations in the intestinal embryo from as early as 16 days of incubation. There is also evidence supporting the vertical transfer of salmonella into the egg (Gantos et al., 2009), a longitudinal study demonstrating vertical transmission of *E. coli* from broiler breeders to broilers (Poulsen et al., 2017), and more recently, the finding of maternal oviduct microbiota in subsequent embryo ceca bacterial populations (Lee et al., 2019). Stanley et al. (2013) indicated that the commercial practices involved with hatching of eggs, including washing and fumigation of eggs, expose chicks to non‐indigenous bacteria at hatch and accordingly heavily influences colonization, which likely accounts for the large inter‐individual variations we see in poultry microbial studies. It is likely that genetic factors are having a much greater effect however than once perceived, supported by the differing microbial phylotypes in the absence of environmental confounding, including the hatchery, within the current study.

The microbial community structure significantly differed between the three groups of birds at phylum, genus and OTU levels, with clear separation of the strains observed in the redundancy analysis plots. As anticipated *Firmicutes* were the highest represented phylum across samples; however, the high abundance of *Proteobacteria* in the layer and cross birds at d28 was unexpected. At the genus level, this appeared to be driven by high abundance of by *Escherichia,* and mega Blast of the OTU identified the predominate abundance as sequences identical in sequence with E. coli strain 1500. In the first‐week post‐hatch, *Proteobacteria*, in particular *E. coli*, are primary colonizers in the caecum, and generally replaced with families *Lachnospiraceae* and *Ruminococcaceae* (phylum *Firmicutes*), then subsequent colonization by the phylum *Bacteroidetes* (Rychlik, 2019). This gradual colonization of the intestinal microbiota is associated with commercial hatching of birds, as recent studies comparing control chicks vs. chicks co‐raised with hens, showed that the microbial composition of the latter reached a composition similar to that of an adult hen by 5 days post hatch, even after as little as 24 h exposure to the hen (Kubasova et al., 2019). Within the current study, although this slower commercial colonization progression is apparent, there remains a distinct difference in the colonization pattern between strains, supporting a strong genotypic effect. Differing colonization patterns may have management implications, including litter waste management, and targeted modulation of the microbiota separate for broiler and layer production systems.

Previous hepatic transcriptional comparisons of layers, broilers and their F1 cross also found significant differences amongst genes involved in cell cycle regulation and evidence of increased glucose uptake and glycolysis in the liver (Willson et al., 2018). It has been previously shown that the microbiota promotes glucose metabolism at a systemic level and intestinal level (Donohoe et al., 2012). This may be reflected in the differing microbial compositions, and, liver processing of glucose observed between the broiler, layer and F1 cross birds; however, we are limited to draw further correlative conclusions in the current study due to the differing time points of the previous RNA‐seq (d14 post hatch) (Willson et al., 2018) and the current 16S rRNA data (d28 post hatch). Previous analysis of the 155 differentially expressed genes between broiler, F1 cross and layer birds demonstrated significant up or down regulation between the broiler and layer birds and intermediate expression in the F1 cross birds (Willson et al., 2018). This same effect was also evident in the microbial analysis, occurring in six of the eight differentially abundant genera. It is well‐known that host genetic variation drives phenotypic variation, but growing evidence in mice and humans demonstrates that the gut microbiota is also influenced by the genetic state of the host (Goodrich et al., 2014; Lim et al., 2017).

In summary, the microbiota phylotype between broilers, layers and their F1 cross significantly differed in community structure, diversity and relative abundance in the absence of environmental confounding, which is generally difficult to avoid in microbial studies. The results provide a unique comparison and additional evidence that there is a strong genetic component driving microbial composition within poultry strains, despite the embryonic development occurring *in ovo*. The current study was unable to address the effect of maternal environment; however, it is plausible that the effect is significant and contributes to inter‐individual variation. The increased *Proteobacteria* in layer birds, particularly *E. coli,* was unexpected at this time point, and comparisons at later time points would allow investigations as to whether particular strains of poultry are pre‐disposed to carry undesirable bacteria into production systems.

## CONFLICT OF INTEREST

No conflict of interest declared.
